# Lithium diaqua­magnesium *catena*-borodiphosphate(V) monohydrate, LiMg(H_2_O)_2_[BP_2_O_8_]·H_2_O, at 173 K

**DOI:** 10.1107/S160053680801516X

**Published:** 2008-05-24

**Authors:** Jin-Ru Lin, Ya-Xi Huang, Yu-Huan Wu, Yan Zhou

**Affiliations:** a Department of Materials Science and Engineering, College of Materials, Xiamen University, Xiamen 361005, Fujian Province, People’s Republic of China

## Abstract

The crystal structure of LiMg(H_2_O)_2_[BP_2_O_8_]·H_2_O consists of tubular structural units, built from tetra­hedral _∞_^1^{[BP_2_O_8_]^3−^} borophosphate ribbons and (LiO_4_)_*n*_ helices running along [001], which are inter­connected by MgO_4_(H_2_O)_2_ octa­hedra, forming a three-dimensional network structure with one-dimensional channels along [001] in which the water mol­ecules are located. The water mol­ecule in the channel is significantly displaced by up to 0.3 Å from the special position  6*b* (..2) to a half-occupied general position. Mg, B and one Li atom all lie on twofold axes. Of the two Li positions, one is at a special position 6*b* (..2), while the other is at a general position; both are only half-occupied.

## Related literature

For NaMg(H_2_O)_2_[BP_2_O_8_]·H_2_O and KMg(H_2_O)_2_[BP_2_O_8_]·H_2_O, see: Kniep *et al.* (1997 ▶). For LiCu(H_2_O)_2_[BP_2_O_8_]·(H_2_O) and LiZn(H_2_O)_2_[BP_2_O_8_]·H_2_O, see: Boy & Kniep (2001*a* ▶,*b* ▶). For LiCd(H_2_O)_2_[BP_2_O_8_]·H_2_O, see: Ge *et al.* (2003 ▶).

## Experimental

### 

#### Crystal data

LiMg(H_2_O)_2_[BP_2_O_8_]·H_2_O
*M* *_r_* = 286.05
Hexagonal, 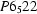

*a* = 9.4139 (1) Å
*c* = 15.7113 (3) Å
*V* = 1205.82 (3) Å^3^
*Z* = 6
Mo *K*α radiation
μ = 0.67 mm^−1^
*T* = 173 (2) K
0.16 × 0.07 × 0.07 mm

#### Data collection

Oxford Diffraction Gemini R Ultra CCD area-detector diffractometer
Absorption correction: numerical (*CrysAlis RED*; Oxford Diffraction, 2005 ▶) *T*_min_ = 0.900, *T*_max_ = 0.954
3404 measured reflections
1343 independent reflections
1142 reflections with *I* > 2σ(*I*)
*R*_int_ = 0.033

#### Refinement

*R*[*F* ^2^ > 2σ(*F* ^2^)] = 0.037
*wR*(*F* ^2^) = 0.092
*S* = 1.06
1343 reflections
75 parameters
H-atom parameters not refined
Δρ_max_ = 0.74 e Å^−3^
Δρ_min_ = −0.55 e Å^−3^
Absolute structure: Flack (1983 ▶), 410 Friedel pairs
Flack parameter: −0.1 (2)

### 

Data collection: *CrysAlis CCD* (Oxford Diffraction, 2005 ▶); cell refinement: *CrysAlis CCD*; data reduction: *CrysAlis RED* (Oxford Diffraction, 2005 ▶); program(s) used to solve structure: *SHELXS97* (Sheldrick, 2008 ▶); program(s) used to refine structure: *SHELXL97* (Sheldrick, 2008 ▶); molecular graphics: *DIAMOND* (Brandenburg, 1997-2004 ▶) and *ATOMS* (Dowty, 2004 ▶); software used to prepare material for publication: *SHELXL97*.

## Supplementary Material

Crystal structure: contains datablocks I, global. DOI: 10.1107/S160053680801516X/br2074sup1.cif

Structure factors: contains datablocks I. DOI: 10.1107/S160053680801516X/br2074Isup2.hkl

Additional supplementary materials:  crystallographic information; 3D view; checkCIF report

## Figures and Tables

**Table:** 

P1—O3	1.501 (2)
P1—O4	1.5033 (19)
P1—O1^i^	1.558 (2)
P1—O2^ii^	1.5632 (17)
Mg1—O4^i^	2.042 (2)
Mg1—O3^iii^	2.0590 (17)
Mg1—O5	2.179 (2)
B1—O1^ii^	1.461 (3)
B1—O2^iv^	1.469 (3)
Li1—O3^v^	2.113 (12)
Li1—O5^v^	2.135 (2)
Li1—O6^vi^	2.35 (2)
Li2—O6^vii^	1.82 (2)
Li2—O4^vii^	2.08 (2)
Li2—O6^viii^	2.08 (2)
Li2—O5^ix^	2.10 (2)

**Table:** 

B1^x^—O1—P1^x^	128.00 (16)
B1—O2—P1^ii^	130.40 (17)
P1—O3—Mg1	130.05 (11)

Symmetry codes: (i) 
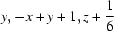
; (ii) 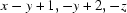; (iii) 
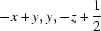
; (iv) 
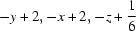
; (v) 
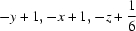
; (vi) 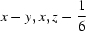; (vii) 
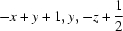
; (viii) 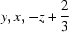; (ix) 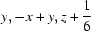; (x) 
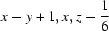
.

**Table 2:** Hydrogen-bond geometry (Å, °)

*D*—H⋯*A*	*D*—H	H⋯*A*	*D*⋯*A*	*D*—H⋯*A*
O5—H1⋯O4^iii^	0.86	2.09	2.852 (3)	148
O5—H2⋯O2^xi^	0.88	1.98	2.779 (3)	151
O5—H2⋯O3^v^	0.88	2.46	3.198 (3)	142

Symmetry codes: (iii) 
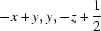
; (v) 
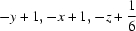
; (xi) 
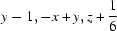
.

**Fig. 1.**
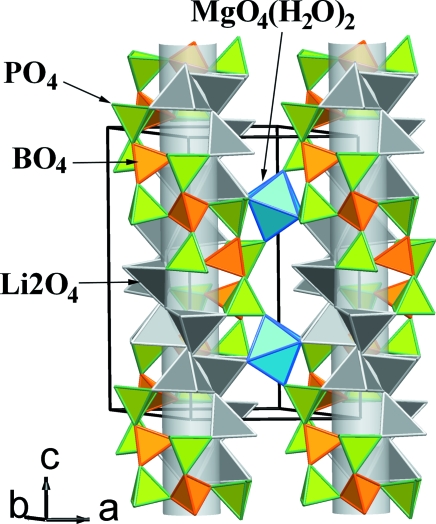
Helical arrangements in the crystal structure of LiMg(H2O)2[BP2O8].H2O, Green tetrahedra: PO4, Orange tetrahedra: BO4, grey tetrahedra: Li2O4.

**Fig. 2.**
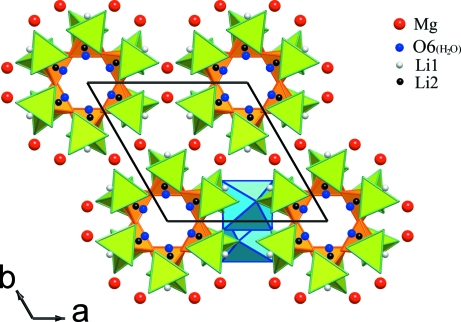
The crystal structure of LiMg(H2O)2[BP2O8].H2O plotted in projection along [001]

**Fig. 3.**
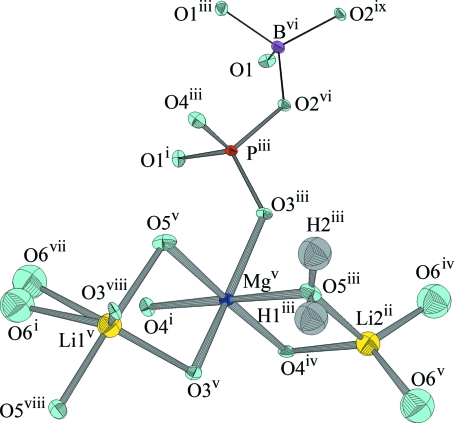
The coordination environment of the metal atoms in LiMg(H2O)2[BP2O8].H2O, with displacement ellipsoids drawn at the 50% probability level.(symmetry codes: (i) y, x, -1/3 - z; (ii) x-y, 1 - y, -z; (iii) x, 1 + x-y, -1/6 - z; (iv) 1 - x,1 - y,-1/2 + z; (v) -x + y, 1 - x, -2/3 + z; (vi) 1 + x-y, x, -1/6 + z; (vii) y, 1 - x + y, -5/6 + z; (viii) -x + y, y, -1/2 - z; (ix) 1 + x-y, 2 - y, -z)

## supplementary crystallographic information

### Comment

In the last decade, much attention has been paid to the large family of borophosphates with the general formula *AM*(H_2_O)_2_[BP_2_O_8_].yH_2_O (A^I^=Li, Na, K, NH_4_^+^; *M*^II^=Mg, Mn, Fe, Co, Ni, Cu, Zn, Cd)(where *y* = 0.5–1) due to their chiral structure property and potential applications for catalysts (Kniep *et al.*, 1997; Ewald *et al.*, 2007). Many combinations between monovalent *A* cations and divalent *M* cations are available. At the *M* site, most of the known compounds in this family apply transition metal ions, while only two magnesium components, NaMg(H_2_O)_2_[BP_2_O_8_].H_2_O as well as KMg(H_2_O)_2_[BP_2_O_8_].H_2_O, are listed for the inclusion of alkaline-earth metals (Kniep *et al.*, 1997). Whereas at the *A* site, up to date, only three Li-based borophosphates are known, *e.g.* LiCu(H_2_O)_2_[BP_2_O_8_].(H_2_O) (Boy & Kniep, 2001*a*), LiZn(H_2_O)_2_[BP_2_O_8_].H_2_O (Boy & Kniep, 2001*b*) and LiCd(H_2_O)_2_[BP_2_O_8_].H_2_O (Ge *et al.*, 2003). Therefore herein we report on a new member of this family with the combination of Li and Mg, LiMg(H_2_O)_2_[BP_2_O_8_].H_2_O.

The crystal structure of the title compound contains infinite one-dimensional helical borophosphate ribbons _∞_^1^{[BP_2_O_8_]^3-^}, arranged around 6_5_ screw axes, which are built up from four-membered rings of corner-sharing PO_4_ and BO_4_ tetrahedra (Fig. 1 & 2). There are two partially occupied Li positions. Li1, located at the outside of ribbons (Fig. 2), is fixed by an irregular arrangement of five oxygen atoms from adjacent phosphate groups (O3) and water molecules (O5, O6). Li2 is tetrahedrally coordinated by four oxygen atoms that originated from phosphate groups (O4) and two water molecules (O5, O6)(Fig. 3). The resulting distorted tetrahedra, (Li2O_4_)_n_, located at the free thread of the borophosphate ribbons, are also wound around 6_5_ screw axes to form helices *via* corner-sharing oxygen atoms which further connect the above infinite one-dimensional borophosphate ribbons by sharing common oxygen corners to develop into a tubular structure where water molecules (O6) with a disorder mode reside in. The magnesium site is octahedrally coordinated by four oxygen atoms from phosphate tetrahedra (O3, O4) and two water molecules (O5) which are located between the adjacent tubes and connect the neighbouring tubes to form a three-dimensional framework.

### Experimental

Transparent, colorless single crystals of the title compound were hydrothermally synthesized. A mixture of MgCl_2_.6H_2_O (0.5344 g), LiOH.H_2_O (5.042 g), H_3_BO_3_ (1.568 g) and 10 ml (85%) H_3_PO_4_ in an approximate molar ratio Mg:Li:B:*P* = 1:46:10:65, are dissolved in 5 ml distilled water while stirring. The resulting solution (pH = 1.5) was transferred into a Teflon-lined autoclave (internal volume 30 ml, degree of filling 67%) and held at 463 K for four days under autogenous pressure. Then the autoclave was cooled to room temperature by turning off the power. Products were filtered off, washed with distilled water and dried at room temperature. Crystals with hexagonal bipyramidal morphology were selected for single-crystal diffraction after checking under a polarizing microscope and identifying by X-ray powder diffraction.

### Refinement

The hydrogen atoms connected with O5 are located from the difference Fourier maps and fixed the positions and displacement parameters assigned as 0.05. The hydrogen positions for O6 are not determined due to the connecting water molecule in disorder mode. The occupancy of O6 was fixed to 0.5 in the last cycles of refinement because its refined value was close to 50%.

### Crystal data

**Table:**

LiMg(H_2_O)_2_[BP_2_O_8_]·H_2_O	*Z* = 6
*M**_r_* = 286.05	*F*_000_ = 864
Hexagonal, *P*6_5_22	*D*_x_ = 2.364 Mg m^−^^3^
Hall symbol: P 65 2 (0 0 1)	Mo *K*α radiation λ = 0.71073 Å
*a* = 9.41390 (10) Å	Cell parameters from 3404 reflections
*b* = 9.41390 (10) Å	θ = 2.8–32.6º
*c* = 15.7113 (3) Å	µ = 0.67 mm^−^^1^
α = 90º	*T* = 173 (2) K
β = 90º	Hexagonal bipyramidal, colourless
γ = 120º	0.16 × 0.07 × 0.07 mm
*V* = 1205.82 (3) Å^3^	

### Data collection

**Table:**

Oxford Diffraction CCD area-detector diffractometer	1343 independent reflections
Radiation source: fine-focus sealed tube	1142 reflections with *I* > 2σ(*I*)
Monochromator: graphite	*R*_int_ = 0.033
*T* = 173(2) K	θ_max_ = 32.6º
326 images,Δω=1°, Exp time: 40 s. scans	θ_min_ = 2.8º
Absorption correction: numerical(CrysAlis RED; Oxford Diffraction, 2005)	*h* = −7→14
*T*_min_ = 0.900, *T*_max_ = 0.954	*k* = −13→14
3404 measured reflections	*l* = −22→8

### Refinement

**Table:**

Refinement on *F*^2^	Hydrogen site location: inferred from neighbouring sites
Least-squares matrix: full	H-atom parameters not refined
*R*[*F*^2^ > 2σ(*F*^2^)] = 0.037	*w* = 1/[σ^2^(*F*_o_^2^) + (0.0493*P*)^2^] where *P* = (*F*_o_^2^ + 2*F*_c_^2^)/3
*wR*(*F*^2^) = 0.092	(Δ/σ)_max_ < 0.001
*S* = 1.06	Δρ_max_ = 0.74 e Å^−^^3^
1343 reflections	Δρ_min_ = −0.55 e Å^−^^3^
75 parameters	Extinction correction: none
Primary atom site location: structure-invariant direct methods	Absolute structure: Flack (1983), 410 Friedel pairs
Secondary atom site location: difference Fourier map	Flack parameter: −0.1 (2)

### Special details

**Table:**

Geometry. All e.s.d.'s (except the e.s.d. in the dihedral angle between two l.s. planes) are estimated using the full covariance matrix. The cell e.s.d.'s are taken into account individually in the estimation of e.s.d.'s in distances, angles and torsion angles; correlations between e.s.d.'s in cell parameters are only used when they are defined by crystal symmetry. An approximate (isotropic) treatment of cell e.s.d.'s is used for estimating e.s.d.'s involving l.s. planes.
Refinement. Refinement of *F*^2^ against ALL reflections. The weighted *R*-factor *wR* and goodness of fit *S* are based on *F*^2^, conventional *R*-factors *R* are based on *F*, with *F* set to zero for negative *F*^2^. The threshold expression of *F*^2^ > σ(*F*^2^) is used only for calculating *R*-factors(gt) *etc*. and is not relevant to the choice of reflections for refinement. *R*-factors based on *F*^2^ are statistically about twice as large as those based on *F*, and *R*- factors based on ALL data will be even larger.

### Fractional atomic coordinates and isotropic or equivalent isotropic displacement parameters (Å^2^)

**Table:**

	*x*	*y*	*z*	*U*_iso_*/*U*_eq_	Occ. (<1)
P1	0.61413 (8)	0.82894 (8)	0.08573 (4)	0.00616 (14)	
Mg1	0.44657 (8)	0.89313 (15)	0.2500	0.0080 (3)	
O1	0.8112 (2)	0.7888 (2)	−0.06431 (11)	0.0096 (4)	
O2	0.7643 (2)	1.1783 (2)	0.01261 (10)	0.0074 (4)	
O3	0.4841 (2)	0.8559 (2)	0.12481 (11)	0.0113 (4)	
O4	0.6190 (3)	0.6808 (2)	0.11749 (11)	0.0116 (4)	
O5	0.1958 (3)	0.7110 (3)	0.21628 (13)	0.0168 (5)	
B1	0.8496 (2)	1.1504 (2)	0.0833	0.0082 (7)	
O6	0.8943 (11)	0.8068 (7)	0.2666 (4)	0.0528 (18)*	0.50
Li1	0.2395 (13)	0.7605 (13)	0.0833	0.027 (3)*	0.426 (18)
Li2	0.893 (3)	0.750 (3)	0.3456 (13)	0.027 (3)*	0.287 (9)
H1	0.1273	0.6655	0.2575	0.050*	
H2	0.1925	0.6306	0.1868	0.050*	

### Atomic displacement parameters (Å^2^)

**Table:**

	*U*^11^	*U*^22^	*U*^33^	*U*^12^	*U*^13^	*U*^23^
P1	0.0073 (3)	0.0063 (3)	0.0050 (2)	0.0035 (2)	0.0006 (2)	−0.0002 (2)
Mg1	0.0081 (4)	0.0074 (6)	0.0084 (5)	0.0037 (3)	0.0018 (4)	0.000
O1	0.0103 (9)	0.0091 (9)	0.0108 (8)	0.0060 (8)	−0.0029 (7)	−0.0014 (7)
O2	0.0090 (9)	0.0091 (9)	0.0045 (7)	0.0048 (8)	−0.0006 (6)	0.0004 (6)
O3	0.0124 (9)	0.0166 (11)	0.0067 (8)	0.0087 (8)	0.0006 (7)	−0.0030 (7)
O4	0.0186 (10)	0.0074 (9)	0.0098 (7)	0.0073 (8)	−0.0013 (8)	0.0012 (7)
O5	0.0132 (11)	0.0124 (10)	0.0160 (9)	−0.0002 (9)	0.0040 (8)	−0.0044 (8)
B1	0.0100 (14)	0.0100 (14)	0.0065 (15)	0.0063 (16)	−0.0018 (13)	−0.0018 (13)

### Geometric parameters (Å, °)

**Table:**

P1—O3	1.501 (2)	B1—O1^i^	1.461 (3)
P1—O4	1.5033 (19)	B1—O2^ix^	1.469 (3)
P1—O1^i^	1.558 (2)	B1—O2	1.469 (3)
P1—O2^ii^	1.5632 (17)	O6—O6^iii^	0.549 (14)
P1—Li2^iii^	2.94 (2)	Li1—O3^x^	2.113 (12)
Mg1—O4^i^	2.042 (2)	Li1—O5^x^	2.135 (2)
Mg1—O4^iv^	2.042 (2)	Li1—O6^vi^	2.35 (2)
Mg1—O3^v^	2.0590 (17)	Li1—O6^iv^	2.35 (2)
Mg1—O3	2.0590 (17)	Li1—Li2^iv^	2.40 (3)
Mg1—O5	2.179 (2)	Li2—O6^iii^	1.82 (2)
Mg1—O5^v^	2.179 (2)	Li2—O4^iii^	2.08 (2)
Mg1—Li2^vi^	3.07 (2)	Li2—O6^vii^	2.08 (2)
Mg1—Li2^vii^	3.07 (2)	Li2—O5^xi^	2.10 (2)
Mg1—Li1^viii^	3.128 (4)	Li2—Li2^vii^	2.36 (4)
Mg1—Li1	3.128 (4)	Li2—O6^i^	2.48 (2)
B1—O1^ii^	1.461 (3)	Li2—O1^xii^	2.65 (2)
			
O3—P1—O4	115.28 (12)	Li2^iii^—O6—Li2^vii^	116.4 (10)
O3—P1—O1^i^	111.30 (11)	Li2—O6—Li1^xi^	75.9 (9)
O4—P1—O1^i^	105.63 (11)	Li2^iii^—O6—Li1^xi^	69.1 (7)
O3—P1—O2^ii^	106.09 (10)	Li2^vii^—O6—Li1^xi^	114.4 (7)
O4—P1—O2^ii^	111.75 (10)	Li2—O6—Li2^xiii^	117.5 (12)
O1^i^—P1—O2^ii^	106.53 (10)	Li2^iii^—O6—Li2^xiii^	64.5 (10)
O3—P1—Li2^iii^	134.1 (4)	Li2^vii^—O6—Li2^xiii^	142.5 (11)
O1^i^—P1—Li2^iii^	63.9 (4)	Li1^xi^—O6—Li2^xiii^	101.1 (6)
O2^ii^—P1—Li2^iii^	119.2 (4)	O3^x^—Li1—O3	109.7 (9)
O4^i^—Mg1—O4^iv^	95.33 (13)	O3^x^—Li1—O5^x^	80.9 (3)
O4^i^—Mg1—O3^v^	91.45 (8)	O3—Li1—O5^x^	97.7 (4)
O4^iv^—Mg1—O3^v^	99.96 (8)	O3^x^—Li1—O5	97.7 (4)
O4^i^—Mg1—O3	99.96 (8)	O3—Li1—O5	80.9 (3)
O4^iv^—Mg1—O3	91.45 (8)	O5^x^—Li1—O5	177.4 (11)
O3^v^—Mg1—O3	163.06 (13)	O3^x^—Li1—O6^vi^	125.3 (5)
O4^i^—Mg1—O5	178.78 (8)	O3—Li1—O6^vi^	124.4 (5)
O4^iv^—Mg1—O5	85.31 (9)	O5^x^—Li1—O6^vi^	98.0 (6)
O3^v^—Mg1—O5	87.42 (8)	O5—Li1—O6^vi^	84.6 (5)
O3—Mg1—O5	81.04 (8)	O3^x^—Li1—O6^iv^	124.4 (5)
O4^i^—Mg1—O5^v^	85.31 (9)	O3—Li1—O6^iv^	125.3 (5)
O4^iv^—Mg1—O5^v^	178.78 (8)	O5^x^—Li1—O6^iv^	84.6 (5)
O3^v^—Mg1—O5^v^	81.04 (8)	O5—Li1—O6^iv^	98.0 (6)
O3—Mg1—O5^v^	87.42 (8)	O6^vi^—Li1—O6^iv^	13.5 (4)
O5—Mg1—O5^v^	94.06 (13)	O6—Li2—O6^iii^	10.0 (7)
B1^xiii^—O1—P1^xiii^	128.00 (16)	O6—Li2—O4^iii^	121.6 (14)
B1^xiii^—O1—Li2^xii^	147.5 (5)	O6^iii^—Li2—O4^iii^	112.3 (11)
P1^xiii^—O1—Li2^xii^	84.3 (5)	O6—Li2—O6^vii^	92.9 (11)
B1—O2—P1^ii^	130.40 (17)	O6^iii^—Li2—O6^vii^	102.9 (10)
P1—O3—Mg1	130.05 (11)	O4^iii^—Li2—O6^vii^	134.3 (10)
P1—O3—Li1	127.6 (4)	O6—Li2—O5^xi^	121.0 (13)
Mg1—O3—Li1	97.1 (2)	O6^iii^—Li2—O5^xi^	119.2 (11)
P1—O4—Mg1^xiii^	141.40 (12)	O4^iii^—Li2—O5^xi^	86.5 (8)
P1—O4—Li2^iii^	109.2 (6)	O6^vii^—Li2—O5^xi^	101.1 (9)
Mg1^xiii^—O4—Li2^iii^	96.3 (6)	O6—Li2—O6^i^	102.1 (12)
Li2^vi^—O5—Li1	69.3 (8)	O6^iii^—Li2—O6^i^	112.0 (10)
Li2^vi^—O5—Mg1	91.8 (6)	O4^iii^—Li2—O6^i^	125.2 (9)
Li1—O5—Mg1	92.95 (17)	O6^vii^—Li2—O6^i^	9.6 (4)
O1^ii^—B1—O1^i^	102.5 (3)	O5^xi^—Li2—O6^i^	98.5 (8)
O1^ii^—B1—O2	112.10 (10)	O6—Li2—O1^xii^	99.7 (11)
O1^i^—B1—O2	114.14 (10)	O6^iii^—Li2—O1^xii^	98.7 (9)
O1^ii^—B1—O2^ix^	114.14 (10)	O4^iii^—Li2—O1^xii^	60.6 (6)
O1^i^—B1—O2^ix^	112.10 (10)	O6^vii^—Li2—O1^xii^	86.7 (7)
O2—B1—O2^ix^	102.2 (3)	O5^xi^—Li2—O1^xii^	137.7 (9)
Li2—O6—Li2^iii^	144.4 (15)	O6^i^—Li2—O1^xii^	82.4 (6)
Li2—O6—Li2^vii^	84.1 (12)		

Symmetry codes: (i) *y*, −*x*+*y*+1, *z*+1/6; (ii) *x*−*y*+1, −*y*+2, −*z*; (iii) −*x*+*y*+1, *y*, −*z*+1/2; (iv) −*x*+1, −*x*+*y*+1, −*z*+1/3; (v) −*x*+*y*, *y*, −*z*+1/2; (vi) *x*−*y*, *x*, *z*−1/6; (vii) *y*, *x*, −*z*+2/3; (viii) −*x*+*y*, −*x*+1, *z*+1/3; (ix) −*y*+2, −*x*+2, −*z*+1/6; (x) −*y*+1, −*x*+1, −*z*+1/6; (xi) *y*, −*x*+*y*, *z*+1/6; (xii) −*x*+2, −*x*+*y*+1, −*z*+1/3; (xiii) *x*−*y*+1, *x*, *z*−1/6.

### Hydrogen-bond geometry (Å, °)

**Table:**

*D*—H···*A*	*D*—H	H···*A*	*D*···*A*	*D*—H···*A*
O5—H1···O4^v^	0.862	2.085	2.852 (3)	147.93
O5—H2···O2^xiv^	0.875	1.979	2.779 (3)	151.43
O5—H2···O3^x^	0.875	2.463	3.198 (3)	141.96

Symmetry codes: (v) −*x*+*y*, *y*, −*z*+1/2; (xiv) *y*−1, −*x*+*y*, *z*+1/6; (x) −*y*+1, −*x*+1, −*z*+1/6.
